# Identification of the first-in-class dual inhibitors of human DNA topoisomerase IIα and indoleamine-2,3-dioxygenase 1 (IDO 1) with strong anticancer properties

**DOI:** 10.1080/14756366.2022.2140420

**Published:** 2022-11-08

**Authors:** Barbara Kaproń, Anita Płazińska, Wojciech Płaziński, Tomasz Plech

**Affiliations:** a Department of Clinical Genetics, Medical University of Lublin, Lublin, Poland; b Department of Biopharmacy, Medical University of Lublin, Lublin, Poland; c Jerzy Haber Institute of Catalysis and Surface Chemistry, Polish Academy of Sciences, Cracow, Poland; d Department of Pharmacology, Medical University of Lublin, Lublin, Poland

**Keywords:** Antiproliferative activity, docking simulations, immunotherapy, thiosemicarbazide derivatives

## Abstract

Molecular docking of a large set of thiosemicarbazide-based ligands resulted in obtaining compounds that inhibited both human DNA topoisomerase IIα and indoleamine-2,3-dioxygenase-1 (IDO1). To the best of our knowledge, these compounds are the first dual inhibitors targeting these two enzymes. As both of them participate in the anticancer response, the effect of the compounds on a panel of cancer cell lines was examined. Among the cell lines tested, lung cancer (A549) and melanoma (A375) cells were the most sensitive to compounds **1** (IC_50_=0.23 µg/ml), **2** (IC_50_=0.83 µg/ml) and **3** (IC_50_=0.25 µg/ml). The observed activity was even 90-fold higher than that of etoposide, with selectivity index values reaching 125. In-silico simulations showed that contact between **1-3** and human DNA topoisomerase II was maintained through aromatic moieties located at limiting edges of ligand molecules and intensive interactions of the thiosemicarbazide core with the DNA fragments present in the catalytic site of the enzyme.

## Graphical Abstract

**Figure:**
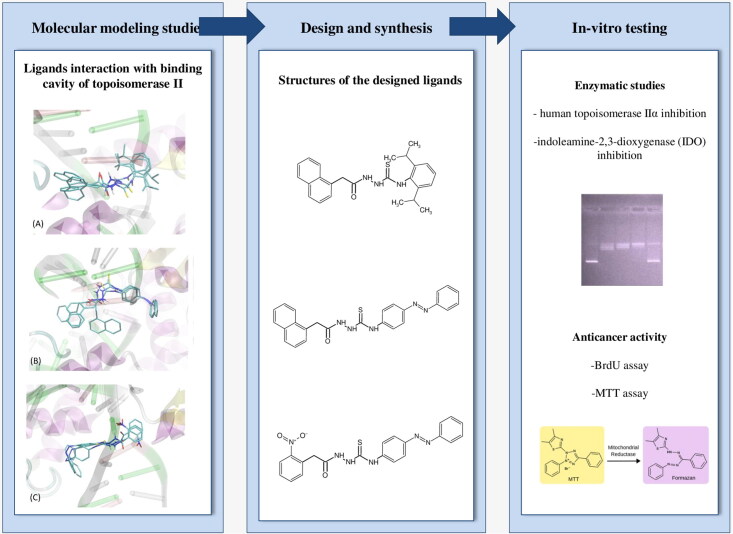

## Introduction

**Figure 1.**
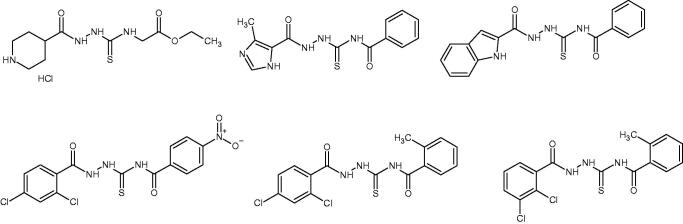
Human topoisomerase Iiα inhibitors based on thiosemicarbazide core^6–8^.

According to WHO report (2020), cancer constitutes one of leading cause of death worldwide^1^. The number of cancer deaths is estimated to be approximately 10 million per year. Only in the USA, about 1.9 million new cases of cancer are expected to be diagnosed in 2022^2^. At the same time, the predicted number of cancer deaths in Europe is approximately 1.2 million^3^, According to National Institute of Health (NIH, USA) overall medical costs associated with cancer care in the USA are projected to reach almost 158 billion of dollars. Further estimations showed that global expenditures for cancer grew to 2.5 trillion of dollars^4^. The above-mentioned epidemiological data confirm that cancer is increasingly a global health-care problem that needs urgent action. From the biological point of view, the basic feature of cancer is uncontrolled growth (proliferation) and spread of abnormal cells from the place of origin to another part of the body. Inhibition of the uncontrolled proliferation is one of the main goal of anticancer therapy. Such effect may be obtained, for example, through the use of chemical compounds that affect replication of DNA in cancer cells. For this reason, human DNA topoisomerase inhibitors (e.g. etoposide, topotecan, irinotecan, mitoxantrone, doxorubicin) are effective anticancer agents^5^. Human DNA topoisomerases catalyse topological changes in single- or double-stranded helices of DNA. These enzymes are necessary in the processes of replication, transcription, recombination, and significantly contribute to the genome stability maintenance^5^. Moreover, since human DNA topoisomerases are directly involved in DNA repair, the application of topoisomerase inhibitors as concomitant drugs during radiotherapy or chemotherapy is one of the strategies enhancing the effectiveness of cancer treatment. In the recent years it turned out that 1,4-disubstituted thiosemicarbazide derivatives have the ability to inhibit human DNA topoisomerases^6–8^ and, subsequently, to reduce the viability of cancer cells (Figure 1). Interestingly, anticancer properties of some of the thiosemicarbazide derivatives have been previously known, but their antiproliferative activity had not been linked to DNA topoisomerase inhibition^9–10^. Therefore, the concept of use of 1,4-disubstituted thiosemicarbazides as anticancer agents acting on the mentioned enzyme is relatively new and not well explored. Much more is known about anticancer properties of thiosemicarbazones, that constitute close analogs of thiosemicarbazides^11^. However, thiosemicarbazones and their metal complexes exhibit antiproliferative activity through the distinct molecular mechanism, that is, through the inhibition of ribonucleotide diphosphate reductase^12–13^. During our previous studies, we identified a group of thiosemicarbazide-based human DNA topoisomerase II inhibitors (Figure 1, lower row) that decreased viability of cancer cells and inhibited intracellular biosynthesis of their DNA much stronger than etoposide – that is, clinically relevant topoisomerase II inhibitor.

Since molecular docking simulations provides insight into the conformation of ligands within the active site of target protein, such approach is an integral part of drug design and discovery. Therefore, in the herein described studies, a large set of 1,4-disubstituted thiosemicarbazides were analysed through the use of molecular docking procedure in order to predict structural features of the most potent inhibitors of human DNA topoisomerase II. Combined application of *in-silico* and *in-vitro* techniques enabled us to identify new inhibitors characterised by strong cytotoxic/antiproliferative properties and high selectivity against cancer cells. Moreover, we have discovered and described the first-in-class dual inhibitors of human DNA topoisomerase II/indoleamine-2,3-dioxygenase 1 (IDO 1) that can lead to the future use of thiosemicarbazide derivatives as relevant components of anticancer immunotherapy.

## Results and discussion

### Design and synthesis of thiosemicarbazide-based inhibitors of human DNA topoisomerase II

The analysed dataset of ligands included molecules composed of thiosemicarbazide skeleton enriched with structurally diverse R1 and R2 substituents (Table 1). Designed ligands had different aryl, aroyl, alkyl (branched and unbranched), heteroaryl groups at distal positions of thiosemicarbazide core (see Supplementary material). The full set of ligands was docked to a single protein topoisomerase II structure found in the PDB database under entry: 3qx3 (X-ray resolution: 2.16 Å, i.e. the highest available among topoisomerase II structures deposited in the PDB database). The binding energy obtained varied from ca. −6 to ca. −11.8 kcal/mol (vs. −14.4 kcal/mol for native ligand), thus, displaying a broad range of affinities towards the molecular target. The aim of this stage was to identify the potentially most potent compounds, exhibiting the lowest protein-ligand binding free energy. Using this criterion, twelve compounds, characterised by the most favourable binding mode (associated with the lower binding energy), were selected for the synthesis and for further in-vitro experiments (Table 1). Compounds **1-12** were obtained in good yields (70–92%) in a one-step reaction between respective carboxylic acid hydrazides and isothiocyanates using procedure described in the literature and in our previous paper^14^. Due to low solubility in culture media and buffers, compounds **11** and **12** were excluded from further *in vitro* experiments.

**Table 1.** Chemical structures of compounds characterised by the most potent affinity towards human DNA topoisomerase II.

Compound No.	Chemical structure	Free energy of binding [kcal/mol]
**1**	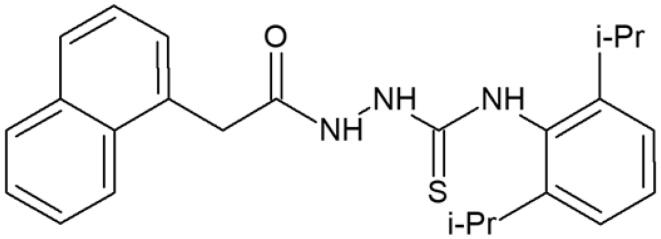	−11.0
**2**	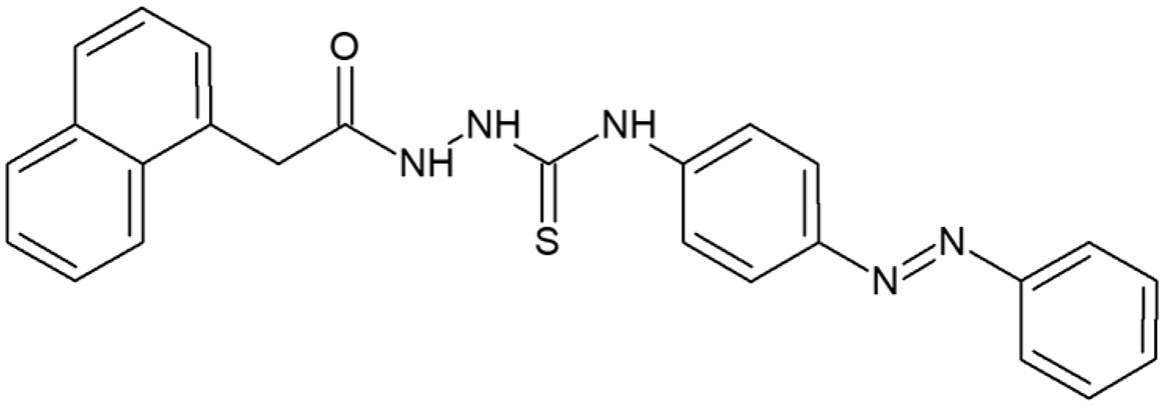	−11.8
**3**	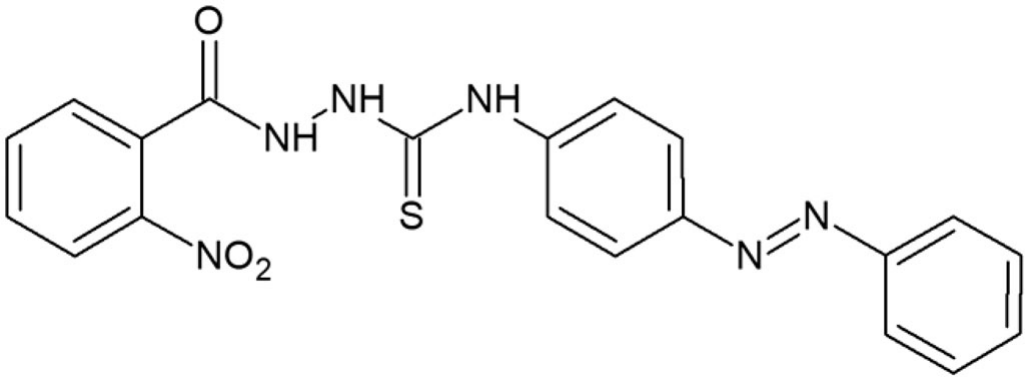	−10.8
**4**	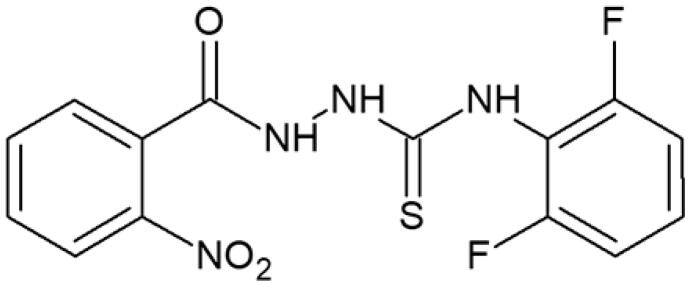	−10.7
**5**	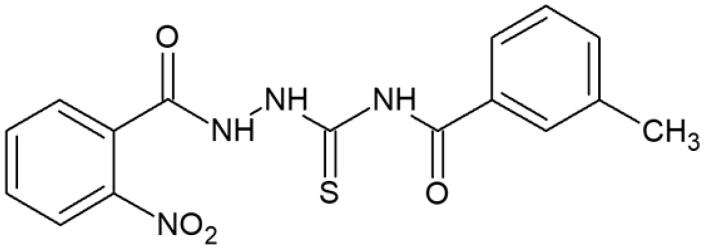	−10.3
**6**	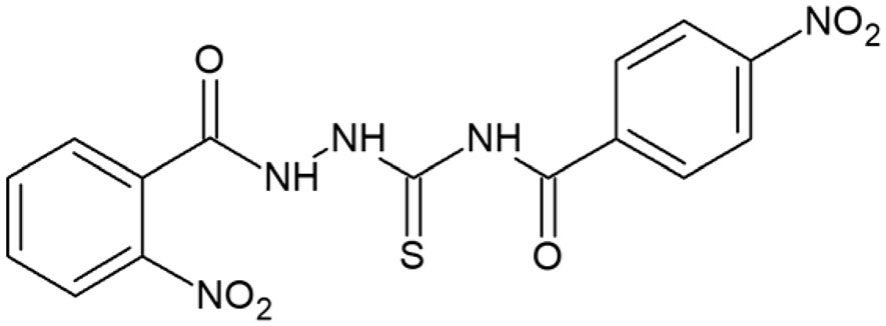	−10.1
**7**	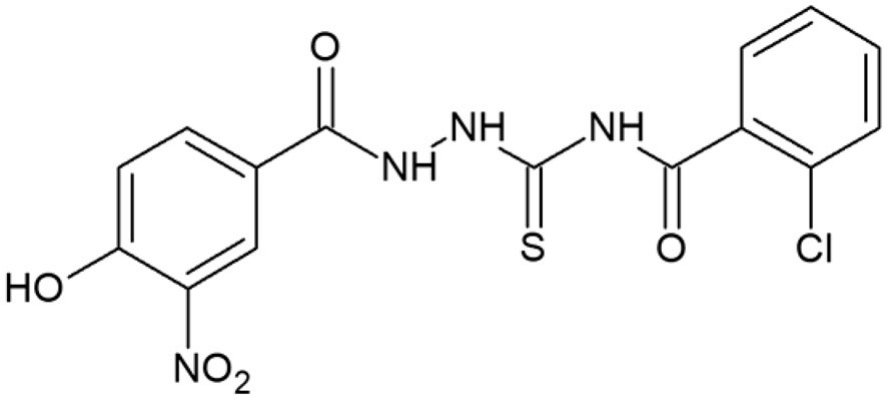	−10.1
**8**	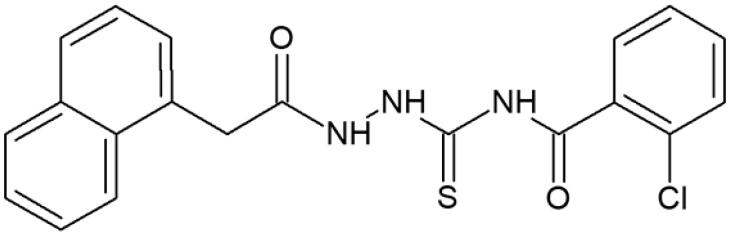	−10.4
**9**	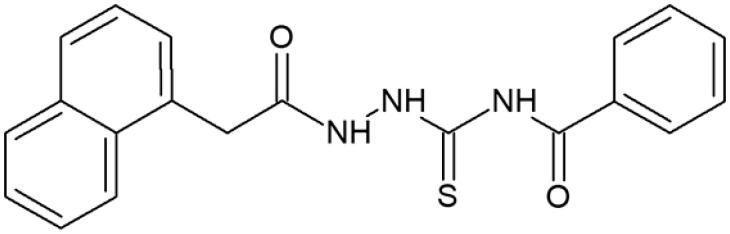	−10.2
**10**	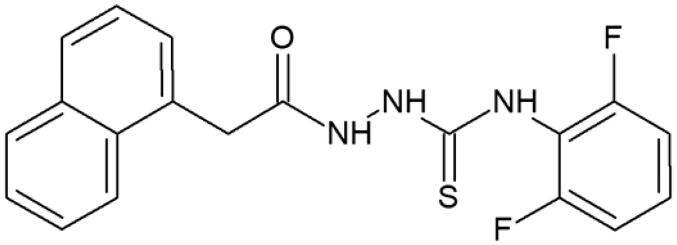	−10.3
**11**	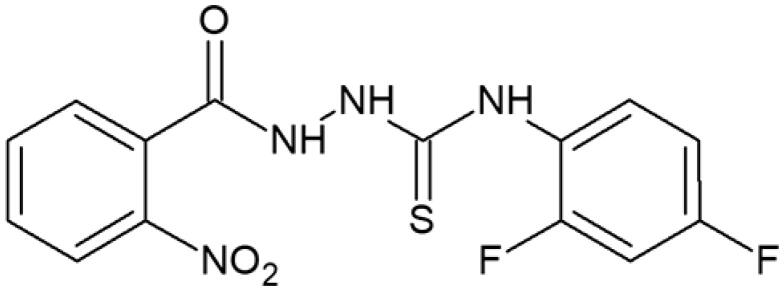	−10.0
**12**	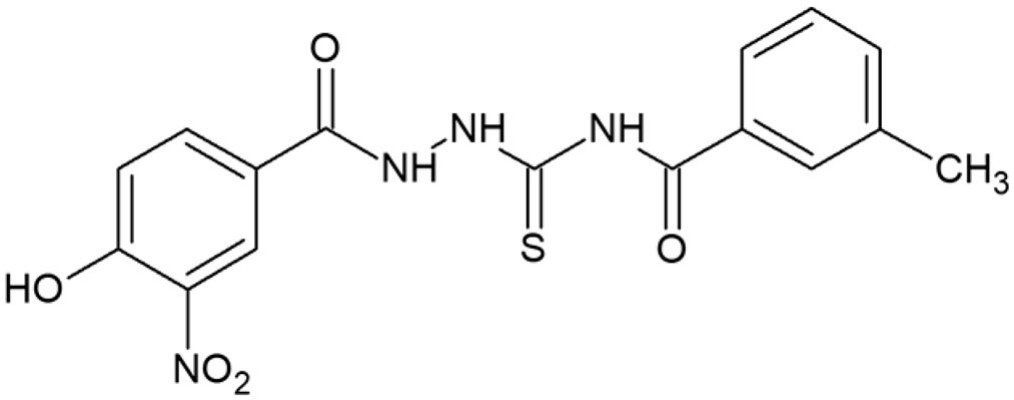	−10.2
**Native ligand**		−14.4

### In vitro testing of compounds 1-10

#### Enzymatic studies

The inhibitory effect of compounds 1-10 and etoposide (positive control) was evaluated using human topoisomerase IIα relaxation assay. As relaxation of supercoiled DNA (scDNA) is one of numerous functions of topoisomerase IIα, so the appearance/disappearance of relaxed DNA bands during agarose gel electrophoresis can be used to identify compounds that inhibit the enzyme. Among the investigated thiosemicarbazide derivatives, compounds 1–3 turned out to inhibit the activity of human DNA topoisomerase Iiα as indicated by the increased intensity of scDNA band and disappearance of relaxed DNA bands (Figure S2, Figure S3). Compound 2 influenced the enzyme activity in a dose-dependent manner with IC_50_ at ∼43µM (Table 2). The other two compounds (1, 3) exhibited lower inhibitory potency against the enzyme (IC_50_ ∼71 and 76 µM, respectively), however it was still higher than that of etoposide (IC_50_ ∼123µM). Importantly, these results are in line with those obtained from docking simulations, since compound 2 exhibited the most favourable free energy of binding (ΔG of −11.8 kcal/mol). During further enzymatic experiments, we also found that compounds 1-3 share the same mechanism of topoisomerase IIα inhibition with etoposide. Both thiosemicarbazide derivatives and etoposide are able to stabilise the covalent DNA-enzyme cleavage complex, which is manifested by the appearance of linear DNA cleavage intermediate (Figure S4). Therefore, compounds 1-3 were found not only to act as inhibitors of human DNA topoisomerase IIα catalytic activity (i.e. relaxation activity), but were also found to be a topoisomerase IIα poisons.

**Table 2.** Inhibition of human DNA topoisomerase Iiα by compounds **1-3** and etoposide.

	IC_50_ [µM] ± SD
**1**	71.04 ± 4.81
**2**	43.11 ± 2.27
**3**	76.80 ± 4.85
**Etoposide**	123.36 ± 5.42

IC_50_ values represent median inhibitory effects of compounds 1-3 and etoposide against relaxation activity of human DNA topoisomerase IIα. Compounds 4-10 had no inhibitory activity (or had only marginal activity) in this assay.

#### Anticancer activity of the investigated compounds

Since the inhibition of human DNA topoisomerase II should result in decreased proliferation of cancer cells, compounds 1-3 were tested using BrdU assay against a panel of cancer cell lines, including MCF-7 (estrogen*-*dependent human breast cancer cells), MDA-MB-231 (estrogen*-*independent human breast cancer cells), SCC-25 (squamous cell carcinoma of the tongue), FaDu (squamous cell carcinoma of the pharynx), A549 (human lung carcinoma), AGS (human gastric adenocarcinoma), LS-180, HT-29 (colon cancer cell lines), T98G (glioblastoma cells), and A375 (melanoma cells). Human normal skin fibroblasts (CRL-2072) were also used as a reference cell line. The BrdU assay allows quantification of cell proliferation since it measures incorporation of thymidine analogue (BrdU) during DNA replication. The examined compounds exhibited wide spectrum of anticancer activity and they inhibited the growth of all types of cancer cells much stronger than reference drug – etoposide (Table 3). Among the cell lines tested, lung cancer (A549) cells were the most sensitive to compounds **1** (IC_50_=0.23 µg/ml), **2** (IC_50_=0.83 µg/ml) and **3** (IC_50_=0.25 µg/ml). These three compounds exhibited strong antiproliferative activity against A375 melanoma cells as well (IC_50_ from 0.56 to 0.96 µg/ml). What is important, while compounds **2** and **3** showed higher selectivity towards A549 and A375 cells, thiosemicarbazide derivative **1** had relatively constant and strong antiproliferative effect over most of the investigated cell lines. Its activity was even 90-fold higher than that of etoposide, with selectivity index values reaching 75 (vs. human normal skin fibroblasts). However, although the antiproliferative effect caused by **3** was mostly weaker (except for A549 and A375) than observed for **1**, but the former compound was also characterised by lower toxicity against human normal cells. It results in selectivity index approaching 125 and 57, when it comes to lung cancer (A549) and melanoma cells (A375), respectively.

**Table 3.** Antiproliferative activity of compounds **1-3** and etoposide examined in BrdU assay.

	IC_50_ [µg/mL] ± SD			
	Etoposide	1	2	3
**MDA-MB-231**	>100	26.29 ± 0.99	>100	>100
**MCF-7**	54.94 ± 1.82	1.19 ± 0.04	33.16 ± 1.05	32.54 ± 1.98
**SCC-25**	21.21 ± 0.74	1.10 ± 0.02	1.06 ± 0.04	4.89 ± 0.22
**A549**	2.60 ± 0.11	0.23 ± 0.01	0.83 ± 0.03	0.25 ± 0.01
**AGS**	45.99 ± 2.61	0.51 ± 0.02	62.34 ± 3.04	43.92 ± 1.94
**T98G**	29.51 ± 0.93	0.72 ± 0.02	6.19 ± 1.83	6.28 ± 0.37
**LS180**	>100	4.68 ± 0.17	10.39 ± 0.77	10.56 ± 0.24
**FaDu**	12.82 ± 0.27	1.04 ± 0.04	7.11 ± 0.34	9.04 ± 0.39
**A375**	9.18 ± 0.15	0.96 ± 0.03	0.85 ± 0.04	0.56 ± 0.03
**HT-29**	19.93 ± 0.25	0.44 ± 0.03	9.47 ± 0.51	13.14 ± 0.81
**Human skin fibroblasts**	55.69 ± 3.01	22.38 ± 0.94	24.17 ± 1.40	32.11 ± 1.75

BrdU incorporation was tested after 24 and 48 h incubation of the cells with the investigated compounds. However, due to strong inhibition of BrdU incorporation by compounds 1-3, the results obtained after 48 h of incubation (IC_50_ values tend to zero for most of the cancer cell lines) were omitted for clarity.

Having in mind that different molecules are able to inhibit the growth of cancer cells through more than one molecular mechanism, it is possible that also other (i.e. other than **1-3**) representatives of the set of synthesised thiosemicarbazide derivatives can exhibit anticancer activity that is unrelated to DNA topoisomerase II inhibition. Therefore, the whole set of compounds were tested by using MTT assay in order to check their overall cytotoxic effect. The growth of cells was monitored after 24 and 48 h of incubation with the increased concentrations (1–100 µg/ml) of the thiosemicarbazide derivatives. The results of MTT assay (Table 4) proved that mainly DNA topoisomerase II inhibition contribute to the anticancer effect of the designed compounds, since compounds **1-3** were characterised by the most potent cytotoxic effect in relation to the other thiosemicarbazide derivatives examined in this study. However, even those compounds that lacked inhibitory effect on DNA topoisomerase II exhibited promising cytotoxic activity against some of the cancer cell lines tested, especially when it was monitored after 48 h of incubation. For example, compound **6** was still up to 10-fold more active than etoposide (against T98G cells), with low cytotoxic properties (IC_50_ > 100 µg/ml) against human normal cells (HEK-293). This confirms that the compounds tested combine different mechanisms of anticancer activity, and DNA topoisomerase II inhibition is only one of them.

**Table 4.** Cytotoxicity of the investigated compounds **1-10** and etoposide against a panel of cancer cell lines measured by using MTT assay after 24 and 48 h incubation.

	IC_50_ [µg/mL]*											
		Etoposide	1	2	3	4	5	6	7	8	9	10
**MDA-MB-231**	24h	>100	9.21	7.64	10.47	>100	>100	>100	>100	>100	>100	83.42
	48h	40.31	8.04	7.07	6.02	32.48	56.31	16.84	69.37	17.23	>100	68.12
**MCF-7**	24h	>100	9.82	9.18	7.67	>100	>100	>100	>100	>100	>100	64.11
	48h	6.15	8.45	8.85	5.48	19.42	28.13	15.93	24.84	12.74	16.37	20.64
**SCC-25**	24h	59.07	7.06	4.73	4.76	>100	>100	25.98	42.41	>100	32.19	52.41
	48 h	23.72	5.22	3.12	3.87	40.86	17.32	9.78	24.32	29.47	34.48	23.86
**A549**	24h	>100	3.87	5.84	5.37	>100	>100	20.77	>100	>100	>100	92.63
	48h	15.59	2.27	5.78	5.13	>100	>100	23.45	>100	>100	>100	32.84
**AGS**	24h	50.79	5.92	5.32	5.54	>100	>100	23.46	>100	>100	>100	29.46
	48 h	29.77	2.69	2.96	4.31	63.87	63.92	29.77	18.76	>100	19.66	21.06
**T98G**	24h	>100	6.98	5.53	3.35	55.47	>100	8.41	11.45	97.22	>100	47.23
	48h	17.06	2.27	4.85	2.94	7.26	30.08	9.78	10.66	11.82	17.39	30.63
**LS180**	24h	>100	4.57	2.87	3.12	>100	>100	>100	>100	>100	>100	72.43
	48 h	11.71	2.54	2.24	2.70	55.62	19.84	10.41	24.68	41.15	19.82	12.55
**FaDu**	24h	12.54	7.26	2.75	2.49	>100	>100	12.89	33.52	>100	28.16	20.12
	48 h	10.73	2.01	2.54	2.18	39.25	31.69	14.77	22.47	12.81	19.53	29.74
**A375**	24h	10.21	4.57	8.96	1.80	>100	89.31	>100	>100	>100	>100	47.92
	48 h	6.48	1.82	2.47	0.56	14.81	38.45	7.98	13.88	9.45	8.14	30.15
**HT-29**	24h	94.49	8.96	8.96	7.37	25.01	45.23	22.78	47.38	>100	>100	32.94
	48 h	11.30	5.33	5.33	3.77	>100	12.30	6.78	7.18	14.77	5.23	32.16
**HEK-293**	24h	37.85	17.60	15.59	27.54	>100	78.14	>100	67.15	>100	67.03	63.71
	48h	29.12	9.61	10.03	9.19	>100	24.59	60.13	32.70	23.42	23.03	19.50

*SD values were omitted for clarity.

The results obtained from MTT assay enabled to conlude that R_2_ substituents play a key role in the anticancer activity of thiosemicarbazide derivatives. It can be observed that bulky substituents (e.g. phenylazophenyl, diisopropylphenyl groups) at N4-position of the thiosemicarbazide core affect topoisomerase II inhibition and cytotoxic properties of the investigated group of compounds. In order to mechanistically explain the primary mode of anticancer action of compounds **1-3**, further *in-silico* simulations were performed.

### Molecular modelling studies

**Figure 2.**
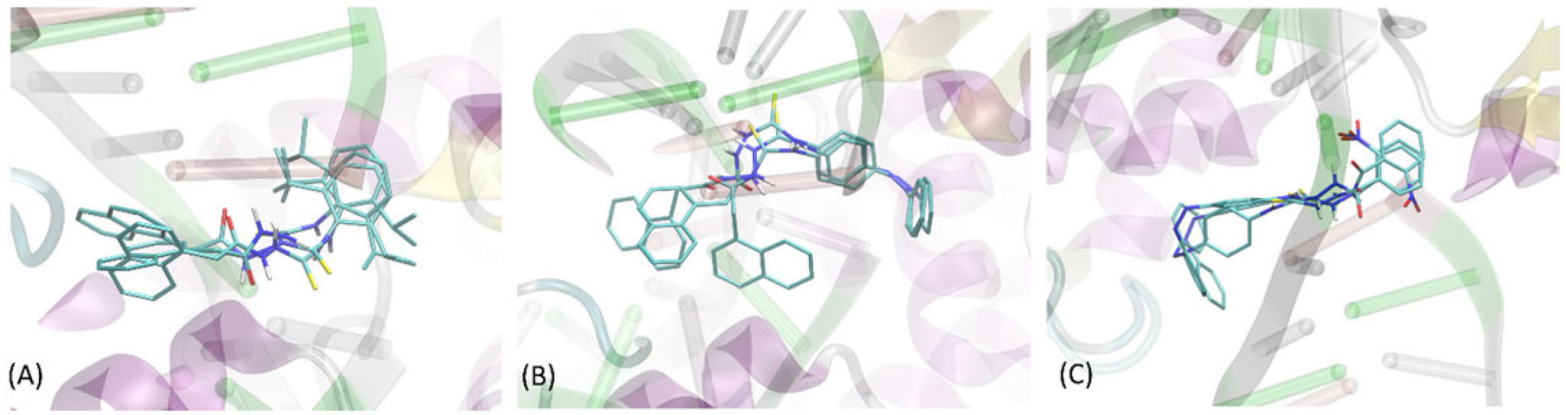
The exemplary, representative poses of the three ligands interacting with binding cavity of topoisomerase II. All depicted poses, characteristic of a given ligand, exhibit similar level of binding energies (see discussion in the text). (A) compound 1; (B) compound 2; (C) compound 3.

Three ligands (i.e. compounds **1-3**), characterised by exceptionally high binding energies as well as by the favourable experimentally determined properties were docked to 6 further (apart from PDB:3qx3) protein structures of topoisomerase II, found in the following PDB entries: 4g0u, 4g0v, 4j3n, 5gwi, 5gwj and 5gwk of resolutions of 2.70, 2.55, 2.30, 2.74, 2.57 and 3.15 Å, respectively. These structures were also cleaned by removing the co-crystalized ligands and ions (if present) but the DNA fragments were left unchanged. Note that these structures belong either to topoisomerase IIα or IIβ, however, due to high structural similarity within the region of binding cavity, we decided to use both those subtypes. Moreover, the high number of structures used in this stage of the study allowed to account for the inherent flexibility of the DNA strand, exhibiting tight contact with the ligand, according to the available XRD data. Figure 2 shows the typical conformational arrangements found for ligands **1-3** interacting with binding cavity. The most frequent pattern of interactions involves: (1) location of the ligand molecule in the protein cleavage, i.e. the binding site for DNA strand; (2) interactions of the ligand with two protein domains separated by the above-mentioned cleavage; (3) intensive interactions within the DNA fragments, present in the binding cavity. All these three aspects are characteristic for native ligands, co-crystalized with the protein structures considered in the current study. At the same time, depending on the ligand type-protein structure combination, we observed significant scatter of possible conformational arrangements of ligand molecules in the binding cavity.

Apart from representative poses shown in Figure 2, alternative poses appeared, differing by the opposite orientation of the ligand in the cavity or interactions with only one site of protein cleavage (and the DNA strand). Although the presence of oppositely oriented poses is expected due to roughly symmetrical structure of ligands (aromatic moieties located at both edges of molecules), the interactions with only one site of catalytic cleavage is contradictory with the available structural data and the knowledge about the mechanism of action of topoisomerases. For this reason (and due to notably higher binding energies), such alternative poses were not analysed in detail. The order of average binding energies (averaged over all seven protein structures of topoisomerase II) obtained for the three lead compounds varies in a rather minor range as follows: −9.83 ± 0.47 (compound 2); −9.77 ± 0.53 (compound 3); −9.23 ± 0.46 kcal/mol (compound 1). This suggests similar potency of all compounds for binding to DNA topoisomerase II.

**Figure 3.**
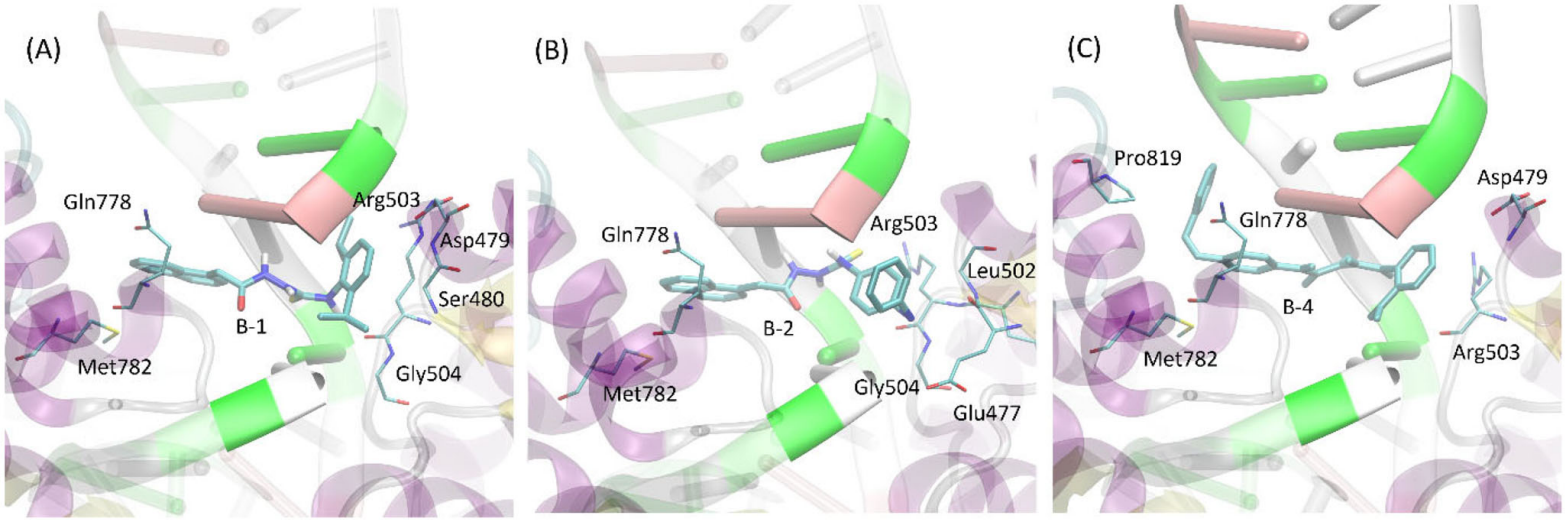
The exemplary, energetically-favourable poses of the three lead compounds identified during docking study: (A) compound **1**; (B) compound **2**; (C) compound **3**.The ligand molecules are shown as thick sticks whereas all the closest amino-acid residues (within the distance of 0.4 nm) are represented by thin sticks. The description of the interaction types is given in the text.

The results of the docking studies have also been analysed with respect to the mechanistic interaction patterns that may be significant in the context of interpretation of the ligand-protein affinities. The summary given below relies on analysing the ligand-protein contacts that occur if the distance between any corresponding atom pair is smaller than the arbitrarily accepted value of 0.4 nm. When considering the most energetically favourable poses, all the studied compounds dock to the protein-DNA complex in a very similar manner (see Figure 3). The contact with protein is maintained through aromatic moieties located at limiting edges of ligand molecules, as well as through smaller substituent of those groups. In the case of compound **2**, an additional contact site is created by the azo moiety linking the two phenyl rings. Those contacts involve either sidechains of backbone fragments belonging to the two different protein domains located at the two sides of catalytic cleavage. Namely, sidechains of Met782, Gln778 and Pro819 interact with phenyl rings of ligand molecule through CH–π or π–π interactions. Some hydrogen bonding between Gln sidechain and the azo moiety is also possible. At the other side of the cleavage, the remaining aromatic groups of the ligand interact with sidechains of Arg503, Glu477, Asp479 and backbone fragments of Gly504, Ser480 and Leu502. The character of interactions varies from CH-π interactions to hydrogen bonding (involving mainly Arg503). As indicated by the higher number of potential contacts, interactions at this side of the cleavage are most intensive and stabilise the ligand position to larger extent in comparison to its second limiting edge (Figure 3).

Apart from protein-related contacts, all ligands exhibit intensive interactions with the DNA fragments present in all crystal structures of topoisomerase II selected to be used in the docking simulations. Such interactions involve the central part of the ligand molecules, composed of the same chemical motif: -CH_2_-C(=O)-NH-NH-C(=S)-NH-. Here, the attractive character of such contacts is the consequence of either CH-π interactions (involving aromatic nucleobases) or hydrogen bonding with ligand molecule playing a role of either donor or acceptor.

### Screening of indoleamine-2,3-dioxygenase (IDO) inhibition

The concept of use of thiosemicarbazide core as an attractive scaffold in anticancer drug design and development was enriched by the results obtained by Serra et al.^15^ Their studies demonstrated that some of the thiosemicarbazide derivatives highlighted inhibitory effect on indoleamine-2,3-dioxygenase (IDO), which is a promising target for anticancer immunotherapy. The use of IDO inhibitors may be a useful strategy to overcome tumour-induced immunosuppression. Moreover, numerous in-vitro and in-vivo clinical trials confirmed that combined application of IDO 1 inhibitors with classical chemotherapy or radiotherapy improved the outcomes of the treatment^16^. Taking into consideration that dual inhibitors, acting both on human topoisomerase II and IDO1, could possess beneficial anticancer properties, the final stage of the study aimed to check if the compounds **1-3** are able to inhibit these two enzymes simultaneously.

Preliminary studies demonstrated that compound **1** has almost the same inhibitory activity against IDO 1 as that of epacadostat, which is very promising anticancer drug candidate (Table 5). The other two thiosemicarbazide derivatives (**2, 3**) were also found as IDO 1 inhibitors, however with relatively lower inhibitory potency. As far as anticancer properties of human DNA topoisomerase inhibitors (e.g. etoposide, topotecan, irinotecan, mitoxantrone, doxorubicin) are well known, contribution of IDO 1 to anticancer effect has been undiscovered for quite a long time. Indoleamine-2,3-dioxygenase (IDO) belongs to the group of cytoplasmic enzymes catalysing the degradation process of tryptophan. As a result of this enzymatic reaction, compounds called kynurenines are formed, which include N-formylkynurenine, L-kynurenine, kynurenine acid, 3-hydroxykynurenine, 3-hydroxyanthranilic acid, quinolinic acid and others. The final effect of these changes is the oxidised form of nicotinamide adenine dinucleotide (NAD^+^). For many years, the IDO enzyme was only attributed to participation in cellular energy processes. However, IDO, and in particular IDO-1, inhibits the immune system and leads to a state of tolerance to specific antigens. Unfortunately, the same mechanism that allows to protect human organism before the development of autoimmune diseases is used by the cancer to develop and to metastasise in the human body. IDO 1 is overexpressed by tumour cells to escape from a potentially effective immune response. Excessive degradation of tryptophan within the tumour microenvironment leads to immunosuppression and inhibit the function of T lymphocytes and NK cells, mainly due to GCN2 kinase and as a result of inhibition of mTOR signaling^17^^,^^18^. The result is reduced proliferation of T lymphocytes and their transition into a state of anergy, which causes them to lose their ability to destroy cancer cells. In addition, inhibition of the mTOR pathway leads to the differentiation of T lymphocytes towards regulatory cells that suppress the immune response. Because IDO is involved in the process of tumour growth, high hopes are associated with the use of small molecule inhibitors of IDO. Currently, the effectiveness of IDO inhibitors, for example, epacadostat, indoximod, navoximod, is being tested at various stages of clinical trials^19^. In preclinical models, inhibition of the IDO 1 has been shown to enhance the efficacy of cytotoxic chemotherapy, radiotherapy and immunotherapy without increasing side effects. Importantly, immunotherapy has come to be a dominant treatment option in melanoma and lung cancer^20^^,^^21^, while the herein described compounds **1-3** possess superior antiproliferative activity against those two types of cancer. Therefore, the above-mentioned dual inhibitors of human DNA topoisomerase II and IDO 1 pave the way to use such thiosemicarbazide derivatives as an effective anticancer strategy to be explored more in further preclinical and clinical studies.

**Table 5.** Inhibitory effect of compounds **1-3** against indoleamine-2,3-dioxygenase 1 (IDO 1).

	IDO 1 inhibition (%) ± SD
**1**	91.37 ± 1.82
**2**	41.37 ± 2.47
**3**	21.43 ± 1.05
**Epacadostat**	99.58 ± 0.43

The compounds were tested in a fixed dose of 50 µg/ml. Since compounds 1-3 were devoid of inhibitory effect towards IDO 2 and TDO (also examined during the assay), therefore these results are not included in the table.

## Conclusions

Summarising, three novel and potent dual inhibitors of human DNA topoisomerase II and IDO 1 were discovered using computer-aided drug design techniques. The obtained thiosemicarbazide derivatives exhibited a wide spectrum of antiproliferative and cytotoxic properties since they effectively inhibit the growth of numerous cancer cell lines tested with IC_50_ values even up to 90-fold lower than etoposide – clinically used chemotherapeutic agent, and selectivity index values reaching 125. Mechanistic studies showed that inhibition of human topoisomerase II by the investigated compounds is maintained through the contact of the protein with aromatic moieties located at limiting edges of ligand molecules and intensive interactions of the thiosemicarbazide core with the DNA fragments present in the catalytic site of the enzyme.

## Materials and methods

### In silico experiments

**Figure 4.**
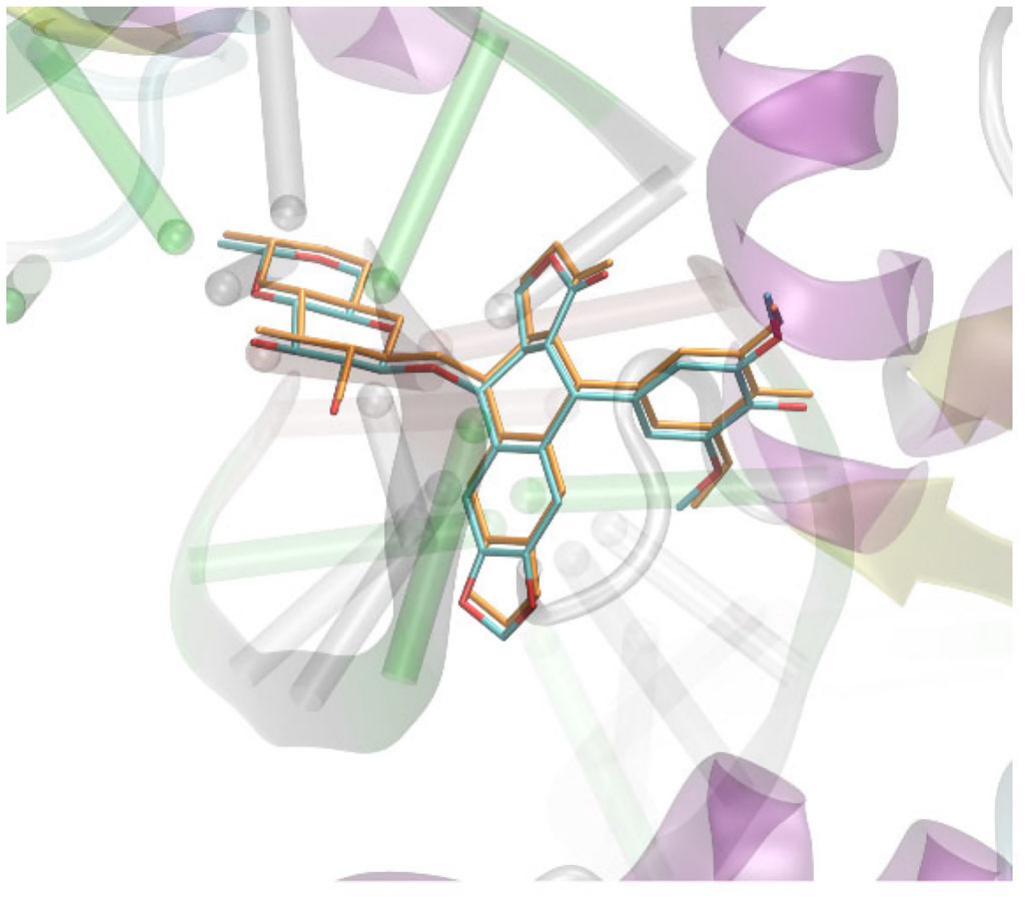
Validation of the docking procedure. The comparison of the position of the ligand present in the crystal structure of protein deposited in PDB:3qx3 (colored by atom type) and the position predicted during docking simulations (colored in orange). Hydrogen atoms are omitted.

A set of approximately 2000 derivatives of thiosemicarbazide, precisely 1,4-disubstituted thiosemicarbazide derivatives, were docked into the DNA-binding sites of human DNA topoisomerase II. Designed ligands had different aryl, aroyl, alkyl (branched and unbranched), heteroaryl groups at distal positions of thiosemicarbazide scaffold. Docking results were ranked on the basis of binding free energies. During in-silico experiments the AutoDockVina software was applied for docking simulations.^22^ All docked molecules were allowed for altering their conformations by introducing rotatable bonds, automatically detected by the prepare_ligand4.py tool of the ATD Tools package. In addition, the flexibility of the following amino-acid residues was allowed: Asp479, Arg503, Gln778 and Met782. The selection of those residues relied on the criterion of contact with the ligand present in the PDB:3qx3 structure. The procedure of docking was carried out within the cuboid region of dimensions of 28 × 28 × 28 Å^3^ which the originally co-crystallised ligands present in the PDB structures as well as the closest amino-acid residues that exhibit contact with those ligands. All the defaults procedures and algorithms implemented in AutoDockVina were applied during docking procedure. The docking methodology was initially validated by docking simulations of the ligand molecule originally included in the protein structure PDB:3qx3. The validation procedure was analogous to that described above for the studied compounds. The graphical illustration of the validation results is given in Figure 4. The accepted methodology is accurate enough to recover the original position of the bound ligand with a small root-mean square deviation of non-hydrogen atoms, equal to 0.0974 Å.

### Chemistry

The selected thiosemicarbazide derivatives, characterised by the most potent affinity towards human DNA topoisomerase II, were synthesised using previously described method^14^. In brief, equimolar amounts of respective acid hydrazides and isothiocyanates were dissolved in anhydrous ethanol and heated under reflux for 10 min. After cooling, the precipitate formed were filtered, dried and crystallised from anhydrous ethanol. Melting temperatures of the synthesised compounds were measured using Fisher-Johns apparatus (Fisher Scientific, Schwerte, Germany). Elemental analyses were determined using AMZ-CHX elemental analyser (PG, Gdansk, Poland) and were within ±0.4% of the theoretical values. NMR spectra were recorded on a Bruker Avance spectrometer (Bruker BioSpin GmbH, Germany) using DMSO-d_6_ as solvent and TMS as an internal standard. FT-IR spectra were recorded using a Vertex 70 spectrometer (Bruker) equipped with an ATR Platinum Diamond A 225 device. All reagents and solvents were purchased from Sigma-Aldrich and were used without further purification.

#### 4-(2,6-Diisopropylphenyl)-1-[(naphth-1-yl)acetyl]thiosemicarbazide (1)

Yield: 74%; m.p.: 140–142 °C; ^1^H-NMR (600 MHz): 1.12 (d, 6H, 2xCH 3, *J* = 6.9 Hz), 1.17 (d, 6H, 2xCH3, *J* = 6.9 Hz), 3.05–3.12 (m, 2H, 2xCH), 4.06 (s, 2H, CH 2), 7.12–8.16 (m, 10H, ArH), 9.19 (s, 1H, NH), 9.49 (s, 1H, NH), 10.30 (s, 1H, NH); ^13^C-NMR (150 MHz): 24.49, 28.11, 37.92, 123.36, 125.26, 125.91, 126.08, 126.31, 127.67, 128.07, 128.38, 128.72, 132.49, 132.80, 133.79, 134.84, 147.15, 170.39, 183.15. IR (ν, cm-1): 3371, 3272, 3016, 2969, 1738, 1518, 1364, 1229; Anal. Calc. for C_25_H_29_N_3_OS (%): C 71.56, H 6.97, N 10.01. Found: C 71.73, H 6.69, N 10.14.

#### 4-(4-Phenylazophenyl)-1-[(naphth-1-yl)acetyl]thiosemicarbazide (2)

Yield: 92%; m.p.: 178–180 °C; 1H-NMR (600 MHz): 4.07 (s, 2H, CH 2), 7.36–8.21 (m, 16H, ArH), 9.52 (s, 1H, NH), 9.92 (s, 1H, NH), 10.34 (s, 1H, NH)) ; ^13^C-NMR (150 MHz): 38.25, 122.91, 125.00, 125.96, 126.17, 126.52, 127.78, 128.50, 128.82, 129.94, 131.73, 132.33, 132.49, 133.80, 142.79, 152.52, 170.60, 183.21; IR (ν, cm-1): 3366, 3336, 3109, 2946, 1738, 1501, 1365, 1216; Anal. Calc. for C_25_H_21_N_5_OS (%): C 68.32, H 4.82, N 15.93. Found: C 68.40, H 4.62, N 15.85.

#### 4-(4-Phenylazophenyl)-1-(2-nitrobenzoyl)thiosemicarbazide (3)

Yield: 87%; m.p.: 168–170 °C; 1H-NMR (600 MHz): 7.24–7.96 (m, 13H, ArH), 9.52 (s, 1H, NH), 9.92 (s, 1H, NH), 10.36 (s, 1H, NH); ^13^C-NMR (150 MHz): 122.90, 124.99, 125.95, 126.19, 126.56, 127.73, 128.47, 128.81, 129.95, 131.73, 132.41, 133.76, 142.78, 152.54, 170.82, 181.42; IR (ν, cm-1): 3366, 3016, 2969, 2946, 1738, 1501, 1365, 1216; Anal. Calc. for C_20_H_16_N_6_O_3_S (%): C 57.13, H 3.84, N 19.99. Found: C 57.20, H 3.72, N 19.81.

#### 4-(2,6-Difluorophenyl)-1-(2-nitrobenzoyl)thiosemicarbazide (4)

Yield: 70%; m.p.: 278–280 °C; 1H-NMR (600 MHz): 7.43–7.55 (m, 4H, ArH), 7.81–7.85 (m, 1H, ArH), 7.90–7.94 (m, 1H, ArH), 8.10–8.14 (m, 1H, ArH), 9.31 (s, 1H, NH), 10.28 (s, 1H, NH), 10.51 (s, 1H, NH); ^13^C-NMR (150 MHz): 124.17, 125.96, 126.13, 126.50, 127.68, 128.29, 128.84, 132.35, 132.50, 133.75, 169.74, 183.15; IR (ν, cm-1): 3348, 3047, 1738, 1536, 1326, 1216. Anal. Calc. for C_14_H_10_F_2_N_4_O_3_S (%): C 47.73, H 2.86, N 15.90. Found: C 47.90, H 2.62, N 15.89.

#### 4-(3-Methylobenzoyl)-1-(2-nitrobenzoyl)thiosemicarbazide (5)

Yield: 80%; m.p.: 194–195 °C; 1H-NMR (600 MHz): 2.40 (s, 3H, CH_3_), 7.37–8.10 (m, 8H, ArH), 11.62 (s, 1H, NH), 11.82 (s, 1H, NH), 12.67 (s, 1H, NH); ^13^C-NMR (150 MHz): 22.06, 124.06, 125.32, 126.11, 126.48, 127.08, 127.86, 132.21, 132.48, 133.76, 134.65, 138.13, 146.73, 162.48, 168.91, 180.03; IR (ν, cm-1): 3345, 3046, 2969, 1738, 1516, 1324, 1216; Anal. Calc. for C_16_H_14_N_4_O_4_S (%): C 53.62, H 3.94, N 15.63. Found: C 53.40, H 3.90, N 15.81.

#### 1-(2-Nitrobenzoyl)-4-(4-nitrobenzoyl)thiosemicarbazide (6)

Yield: 78%; m.p.: 286–288 °C; ^1^H-NMR (600 MHz): 7.58–7.67 (m, 4H, ArH), 7.93–8.04 (m, 4H, ArH), 11.37 (s, 1H, NH), 11.91 (s, 1H, NH), 12.26 (s, 1H, NH); ^13^C-NMR (150 MHz): 118.55, 118.89, 125.81, 129.06, 130.76, 131.22, 137.93, 138.58, 139.66, 151.99, 163.68, 167.14, 181.21; IR (ν, cm-1): 3221, 3016, 1738, 1529, 1365, 1228. Anal. Calc. for C_15_H_11_N_5_O_6_S (%): C 46.27, H 2.85, N 17.99. Found: C 46.43, H 2.72, N 17.88.

#### 4-(2-Chlorobenzoyl)-1-(4-hydroxy-3-nitrobenzoyl)thiosemicarbazide (7)

Yield: 68%; m.p.: 182–184 °C; ^1^H-NMR (600 MHz): 7.75–7.93 (m, 3H, ArH), 8.12–8.20 (m, 2H, ArH), 8.32–8.38 (m, 2H, ArH), 11.68 (s, 1H, NH), 12.19 (s, 1H, NH), 12.50 (s, 1H, NH), 13.90 ((s, 1H, OH); ^13^C-NMR (150 MHz): 118.50, 118.95, 125.91, 127.96, 128.93, 130.87, 133.32, 133.50, 134.40, 138.16, 139.64, 151.90, 163.64, 166.78, 180.97; IR (ν, cm-1): 3750, 3015, 2970, 1738, 1521, 1348, 1216. Anal. Calc. for C_15_H_11_ClN_4_O_5_S (%): C 45.63, H 2.81, N 14.19. Found: C 45.82, H 2.95, N 14.08.

#### 4-(2-Chlorobenzoyl)-1-[(naphth-1-yl)acetyl]thiosemicarbazide (8)

Yield: 83%; m.p.: 174–176 °C; ^1^H-NMR (600 MHz): 4.15 (s, 2H, CH_2_), 7.41–7.59 (m, 8H, ArH), 7.86 (d, 1H, ArH, *J* = 8.3 Hz), 7.94 (d, 1H, ArH, *J* = 8.3 Hz), 8.19 (d, 1H, ArH, *J* = 8.3 Hz), 11.34 (s, 1H, NH), 12.11 (s, 1H, NH), 12.44 (s, 1H, NH); ^13^C-NMR (150 MHz): 37.53, 124.87, 125.98, 126.21, 126.57, 127.58, 127.84, 128.52, 128.84, 129.70, 130.01, 130.38, 132.10, 132.40, 132.57, 133.81, 134.56, 167.76, 167.91, 176.73; IR (ν, cm-1): 3215, 3016, 2970, 1738, 1495, 1365, 1216. Anal. Calc. for C_20_H_16_ClN_3_O_2_S (%): C 60.37, H 4.05, N 10.56. Found: C 60.45, H 4.21, N 10.43.

#### 4-Benzoyl-1-[(naphth-1-yl)acetyl]thiosemicarbazide (9)

Yield: 86%; m.p.: 164–166 °C; ^1^H-NMR (600 MHz): 4.14 (s, 2H, CH_2_), 7.46–7.58 (m, 6H, ArH), 7.62–7.66 (m, 1H, ArH), 7.85 (d, 1H, ArH, *J* = 8.3 Hz), 7.92–7.96 (m, 3H, ArH), 8.19 (d, 1H, ArH, *J* = 8.3 Hz), 11.31 (s, 1H, NH), 11.74 (s, 1H, NH), 12.71 (s, 1H, NH); ^13^C-NMR (150 MHz): 37.53, 124.86, 125.98, 126.21, 126.57, 127.85, 128.53, 128.83, 128.91, 129.16, 132.14, 132.30, 132.39, 133.60, 133.81, 167.87, 168.49, 177.38; IR (ν, cm-1): 3273, 3058, 3005, 2970, 1738, 1525, 1365, 1229. Anal. Calc. for C_20_H_17_N_3_O_2_S (%): C 66.10, H 4.71, N 11.56. Found: C 66.15, H 4.59, N 11.47.

#### 4-(2,6-Difluorophenyl)-1-[(naphth-1-yl)acetyl]thiosemicarbazide (10)

Yield: 77%; m.p.: 186–188 °C; ^1^H-NMR (600 MHz): 4.02 (s, 2H, CH_2_), 7.11–7.56 (m, 7H, ArH), 7.85 (d, 1H, ArH, *J* = 8.2 Hz), 7.94 (d, 1H, ArH, *J* = 8.2 Hz), 8.11 (d, 1H, ArH, *J* = 8.2 Hz), 9.44 (s, 1H, NH), 10.02 (s, 1H, NH), 10.45 (s, 1H, NH); ^13^C-NMR (150 MHz): 38.18, 112.18, 125.05, 125.94, 126.15, 126.48, 127.76, 128.49, 128.80, 129.38, 132.29, 132.53, 133.80, 158.57, 160.25, 170.35, 183.11; IR (ν, cm-1): 3246, 3056, 2953, 1658, 1525, 1325, 1239. Anal. Calc. for C_19_H_15_F_2_N_3_OS (%): C 61.44, H 4.07, N 11.31. Found: C 61.33, H 4.21, N 11.30.

#### 4-(2,4-Difluorophenyl)-1-(2-nitrobenzoyl)thiosemicarbazide (11)

Yield: 73%; m.p.: 182–184 °C; 1H-NMR (600 MHz): 7.54–8.13 (m, 7H, ArH), 9.66 (s, 1H, NH), 10.23 (s, 1H, NH), 10.89 (s, 1H, NH); ^13^C-NMR (150 MHz): 122.92, 123.45, 124.79, 129.96, 130.40, 131.78, 132.19, 134.08, 142.67, 147.72, 152.49, 165.82, 180.11; IR (ν, cm-1): 3328, 3291, 2969, 1738, 1523, 1502, 1378, 1226. Anal. Calc. for C_14_H_10_F_2_N_4_O_3_S (%): C 47.73, H 2.86, N 15.90. Found: C 47.78, H 2.59, N 15.96.

#### 4-(3-Methylbenzoyl)-1-(4-hydroxy-3-nitrobenzoyl)thiosemicarbazide (B12)

Yield: 70%; m.p.: 190–192 °C; ^1^H-NMR (600 MHz): 2.41 (s, 3H, CH_3_), 7.22–7.50 (m, 5H, ArH), 8.07–8.10 (m, 1H, ArH), 8.50–8.52 (m, 1H, ArH), 11.26 (s, 1H, NH), 11.85 (s, 1H, NH), 12.27 (s, 1H, NH), 13.95 (s, 1H, OH); ^13^C-NMR (150 MHz): 20.01, 119.76, 123.22, 125.67, 126.04, 128.84, 131.44, 133.85, 134.25, 134.69, 136.42, 137.28, 155.67, 162.81, 171.25, 180.77; IR (ν, cm-1): 3726, 3245, 3045, 2920, 1738, 1518, 1255; Anal. Calc. for C_16_H_14_N_4_O_5_S (%): C 51.33, H 3.77, N 14.97. Found: C 51.17, H 3.67, N 15.06.

### Cell culturing

Cytotoxicity of the synthesised compounds were examined against a panel of the cancer cell lines, MCF-7 (estrogen*-*dependent human breast cancer cells), MDA-MB-231 (oestrogen*-*independent human breast cancer cells), SCC- 25 (squamous cell carcinoma of the tongue), FaDu (squamous cell carcinoma of the pharynx), A- 549 (human lung carcinoma), AGS (human gastric adenocarcinoma), LS-180, HT-29 (colon cancer cell lines), T98G (glioblastoma cells), A375 (melanoma cells). Human embryonic kidney (HEK-293) cells and human normal skin fibroblasts (CRL-2072) were used as reference cell lines. The investigated cell lines were obtained from American Type Culture Collection (ATCC; Manassas, VA, USA). A375, MCF-7, MDA-MB-231, T98G cells were cultured in Dulbecco’s Modified Eagle’s Medium (DMEM) (Sigma Aldrich, St. Louis, MO, USA) supplemented with 10% heat inactivated foetal bovine serum (FBS), penicillin (100 U/mL), and streptomycin (100 μg/mL). SCC-25 cells were cultured in 1:1 mixture of Dulbecco’s modified Eagle’s medium and Ham’s F12 Medium (Sigma Aldrich, St. Louis, MO, USA) supplemented with 400 ng/ml hydrocortisone, 10% FBS, penicillin (100 U/mL), and streptomycin (100 μg/mL). A549 and AGS cells were maintained in F-12K Medium (Sigma Aldrich, St. Louis, MO, USA). LS-180 and FaDu cells were cultured in Eagle’s Minimum Essential Medium. HT-29 cells were maintained in McCoy’s 5 A Medium (Sigma Aldrich, St. Louis, MO, USA) supplemented with 10%FBS, penicillin (100 U/mL), and streptomycin (100 μg/mL). All the cells were maintained at 37 °C in a humidified atmosphere of 5% CO_2_.

### Evaluation of antiproliferative and cytotoxic effects of the investigated thiosemicarbazide derivatives

#### MTT assay

The synthesised compounds were dissolved in DMSO in order to obtain stock solutions. At the day of the experiment, the suspension of cells (1 × 10^5^ cells/mL) in the respective medium was applied to a 96-well plate at 100 μL per well. After 24 h of incubation, the medium was removed from the wells and replaced by increasing concentrations of compounds (or etoposide) in medium containing 2% FBS. The control cells were cultured only with a medium containing 2% FBS. Cytotoxicity of DMSO was also checked at concentrations present in respective dilutions of stock solution. After 24 h (or 48 h) of incubation, 15 μL of MTT working solution (5 mg/mL in PBS) was added to each well. The plate was incubated for 3 h. Subsequently, 100 μL of 10% SDS solution was added to each well. Cells were incubated overnight at 37 °C to dissolve the precipitated formazan crystals. The concentration of the dissolved formazan was evaluated by measuring the absorbance at λ = 570 nm, using a microplate reader (Epoch, BioTek Instruments, Inc., Winooski, VT, USA). At least two independent experiments were performed in triplicates. The results of the MTT assay were expressed as mean ± SD. DMSO in the concentrations present in the dilutions of stock solutions did not influence the viability of the tested cells. IC_50_ values of the investigated derivatives and standards were calculated using the IC_50_ calculator.^23^

#### BrdU assay

The BrdU assay was performed to determine the effect of synthesised compounds on the proliferation of cancer cells. BrdU assay indicates the rate of incorporation of 5′-bromo-2′-deoxy-uridine (BrdU) during DNA biosynthesis. In brief, cells were plated in 96-well plates (NUNC, Roskilde, Denmark) at the density of 3 × 10^4^ cells/mL. The next day, cells were treated with thiosemicarbazide derivatives or etoposide (in the increasing concentrations, up to 100 µg/ml) or a fresh culture medium. Cell proliferation was examined after 24 h and 48 h of incubation according to the manufacturer’s protocol (Cell Proliferation ELISA BrdU, Roche Diagnostics GmbH, Penzberg,)Germany).

### Inhibition of human topoisomerase IIα

Determination of the inhibitory effect of compounds **1-10** against human topoisomerase Iiα was performed using Human Topoisomerase II Relaxation Assay (Inspiralis Ltd, Norwich, UK) and Topoisomerase II Drug Screening Kit (TopoGEN Inc., Buena Vista, CO, USA) following the manufacturer’s instructions. Briefly, the increased concentrations of the compounds were mixed with the reaction mixture, incubated according to the protocol and, after reaction termination, the samples were analysed by electrophoresis using 1% agarose gel containing 0.5 mg/mL of ethidium bromide in Tris-Acetate-EDTA (TAE) buffer. Visualisation of the bands was performed using an enhanced chemiluminescence system (Syngene G:BOX Chemi XT4, Cambridge, UK). For the quantitative determination of topoisomerase II inhibition, images were densitometrically scanned.

The inhibition of topoisomerase II was calculate from the equation:

$$\%\;\mathrm{Inhibition}=\frac{\mathrm{Intensity}\;of\;\mathrm{compound}-\mathrm{treated}\;\mathrm{DNA}}{\mathrm{Intensity}\;of\;\mathrm{vehicle}-\mathrm{treated}\;\mathrm{control}\;\mathrm{DNA}}\times 100$$

### Inhibition of indoleamine-2,3-dioxygenase

Inhibitory effects of the investigated compounds were determined using Universal IDO1/IDO2/TDO Inhibitor Screening Assay Kit from BPS Bioscience, Inc. (San Diego, CA, USA). This colorimetric assay is based on the measurement of the ability of IDO1, IDO2 and TDO to convert L-tryptophan into N-formylkynurenine (NFK). The experiments were performed according to the manufacturer’s guidelines. The final concentration of the compounds in the reaction mixture was 50 µg/mL. The amount of NFK was measured spectrophotometrically at 320 nm using Epoch BioTek microplate reader (BioTek Instruments, Inc., Winooski, VT, USA). The samples were run in triplicate and the results were expressed as mean ± SD.

## Supplementary Material

Supplemental Material
