# Supplementary material for: Identification of the first-in-class dual inhibitors of human DNA topoisomerase IIα and indoleamine-2,3-dioxygenase 1 (IDO 1) with strong anticancer properties
Source: J Enzyme Inhib Med Chem. 2022 Nov 8;38(1):192–202. doi: 10.1080/14756366.2022.2140420 (PMC9648375; doi:10.1080/14756366.2022.2140420)
Supplement: Supplemental Material [file IENZ_A_2140420_SM1456.pdf]

Design of the thiosemicarbazide-based ligands

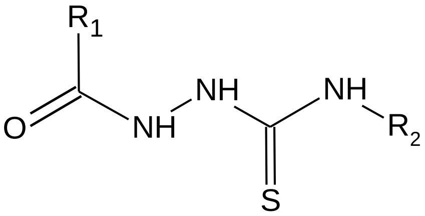

General structure of the ligands

Chemical structures of R1 substituents			
1	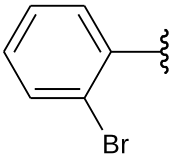	14	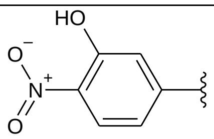
2	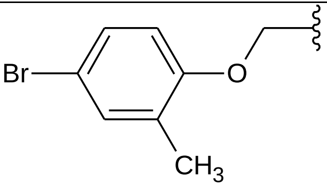	15	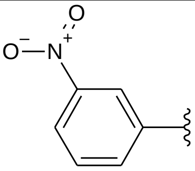
3	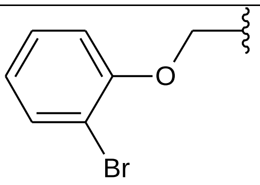	16	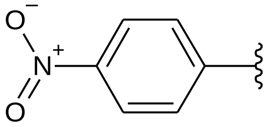
4	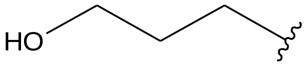	17	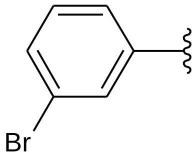
5	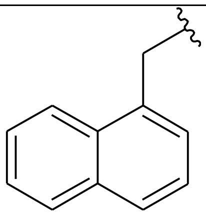	18	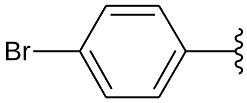
6	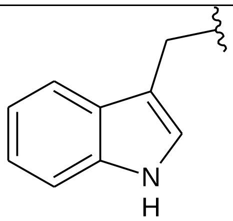	19	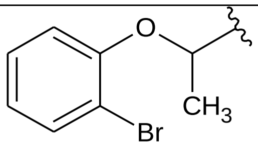

7	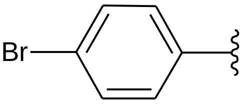	20	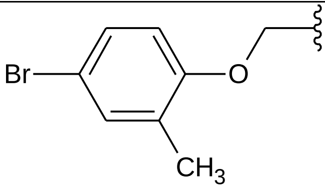
8	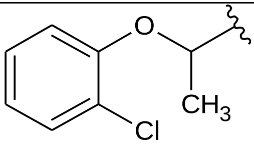	21	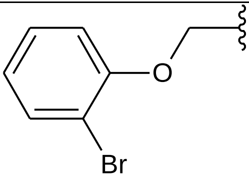
9	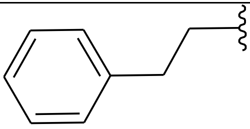	22	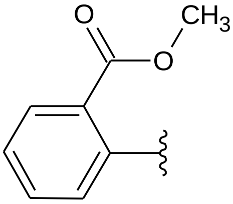
10	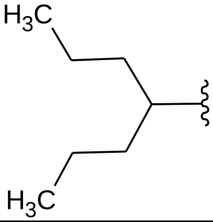	23	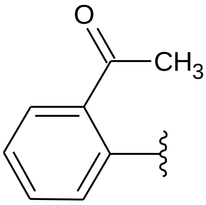
11	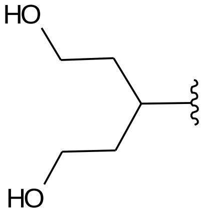	24	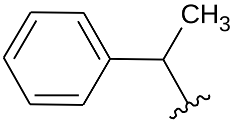
12	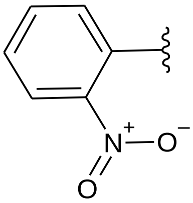	25	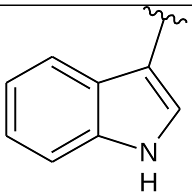
13	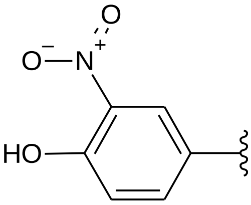		

Chemical structures of R2 substituents			
1	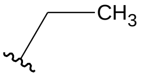	41	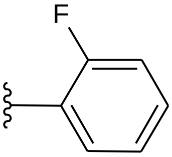
2	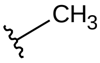	42	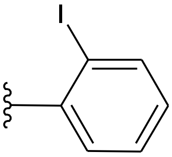
3	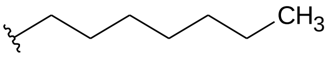	43	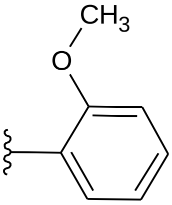
4	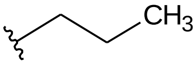	44	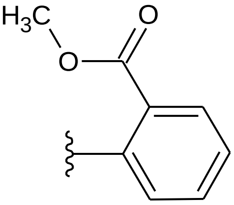
5	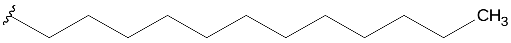	45	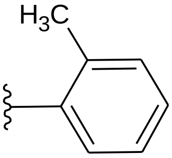
6	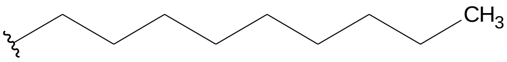	46	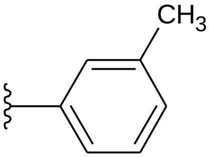
7	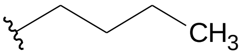	47	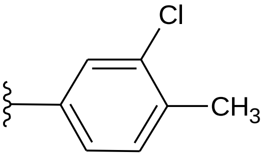
8	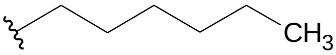	48	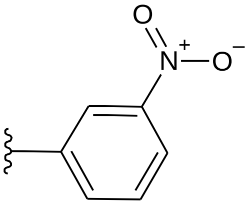

9	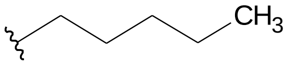	49	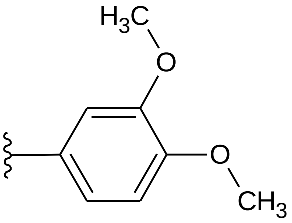
10	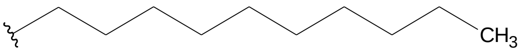	50	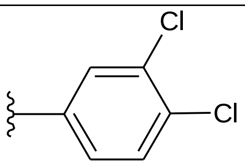
11	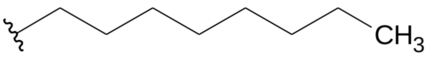	51	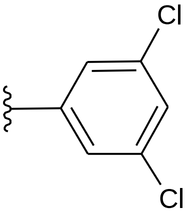
12	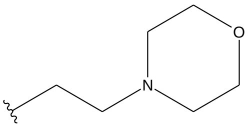	52	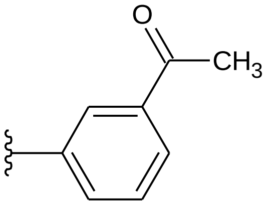
13	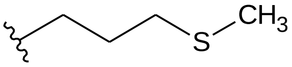	53	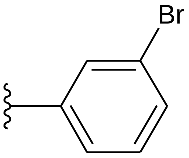
14	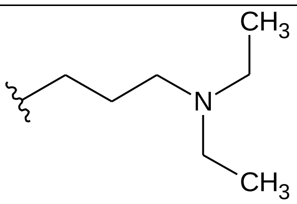	54	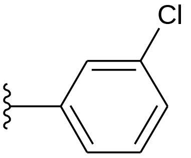
15	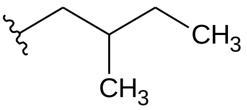	55	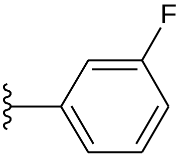
16	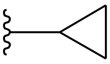	56	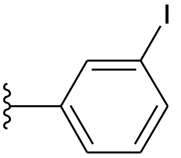
17	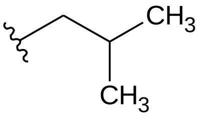	57	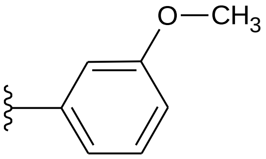

18	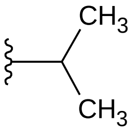	58	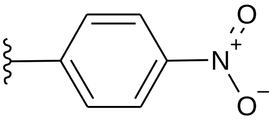
19	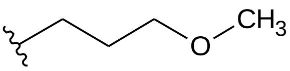	59	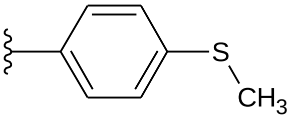
20	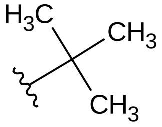	60	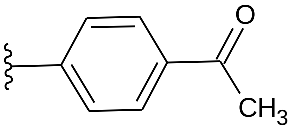
21	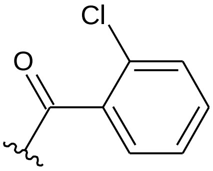	61	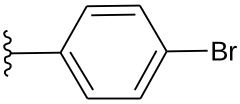
22	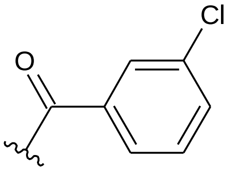	62	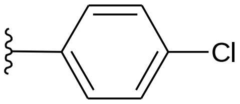
23	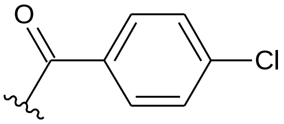	63	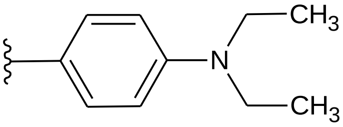
24	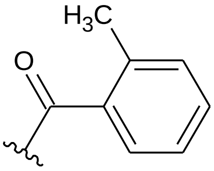	64	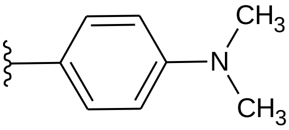
25	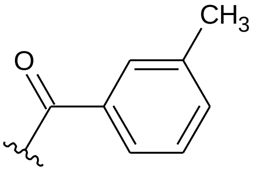	65	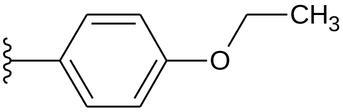
26	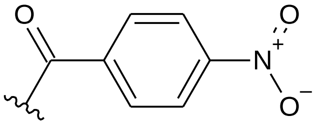	66	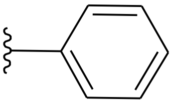
27	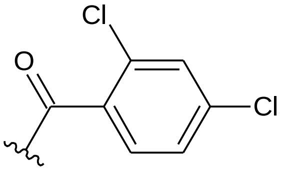	67	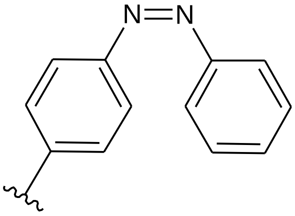

28	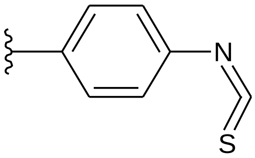	68	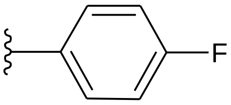
29	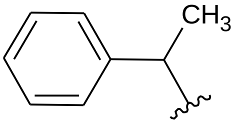	69	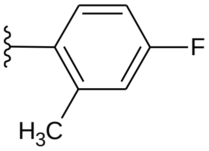
30	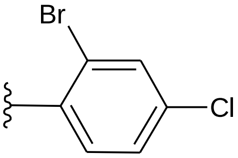	70	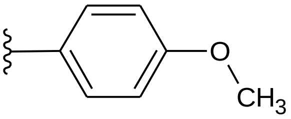
31	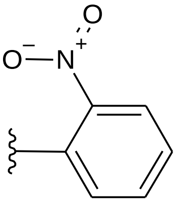	71	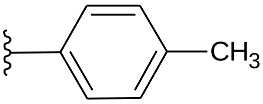
32	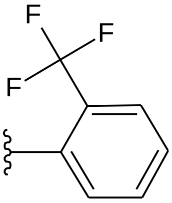	72	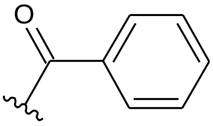
33	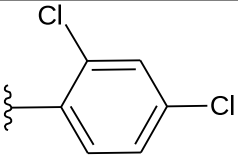	73	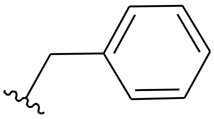
34	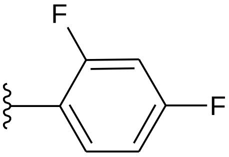	74	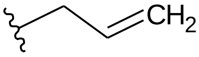
35	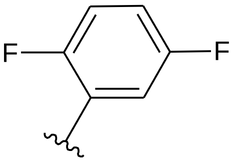	75	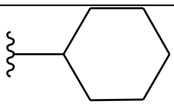

36	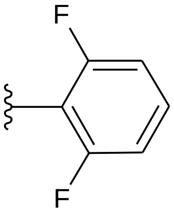	76	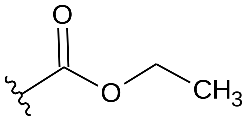
37	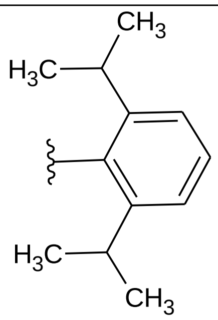	77	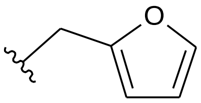
38	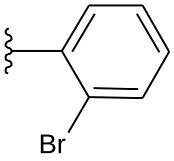	78	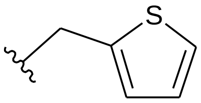
39	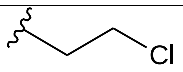	79	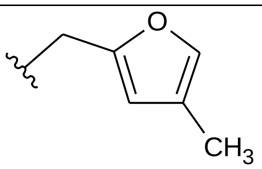
40	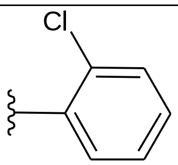	80	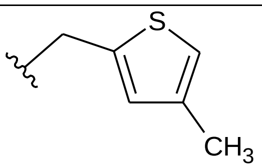

Substitution matrix for the designed ligands

R_1 / R_2	1	2	3	4	5	…	25
1	1-1	2-1	3-1	4-1	5-1	…	25-1
2	1-2	2-2	3-2	4-2	5-2	…	25-2
3	1-3	2-3	3-3	4-3	5-3	…	25-3
4	1-4	2-4	3-4	4-4	5-4	…	25-4
5	1-5	2-5	3-5	4-5	5-5	…	25-5
…	…	…	…	…	…	…	25-…
80	1-80	2-80	3-80	4-80	5-80	…-80	25-80

Explanation: ligand 1-1 contains 2-bromophenyl group as R1 substituent and ethyl group as R2 substituent; ligand 1-2 contains 2-bromophenyl group as R1 substituent and methyl group as R2 substituent; ligand 5-4 contains (naphth-1-yl)methyl group as R1 substituent and n-propyl group as R2 substituent; ....; ligand 25-80 contains 1*H*-indole-3-yl group as R1 substituent and 4-methylthiophene-2-ylmethyl group as R2 substituent.

Ligands characterized by the most potent affinity towards binding site of topoisomerase II structure (PDB id: 3qx3) were renumbered prior to further steps of study, as can be seen in Table 1. For example, compound No. 1 corresponds to ligand No. 5-37.

Enzymatic experiments

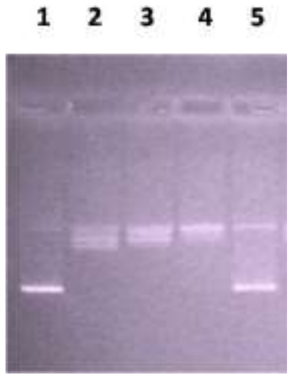

Figure S1. Determination of the inhibitory effect of etoposide in Topoisomerase IIα realaxation assay Lane 1: scDNA; lane 2: scDNA + topoII; lane 3: scDNA + topoII + etoposide (50µM); lane 4: scDNA + topoII + etoposide (100µM); lane 5: scDNA + topoII + etoposide (150µM).

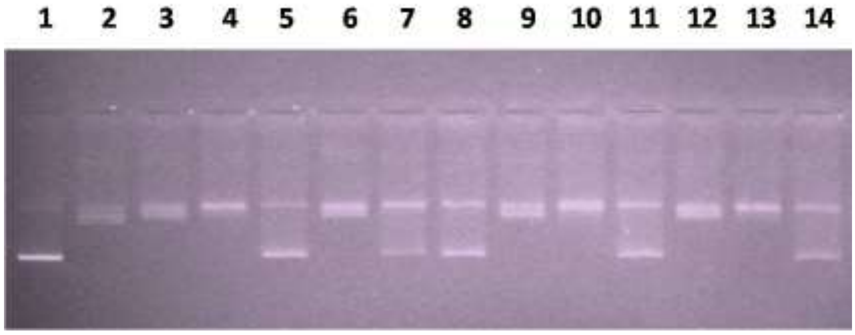

Figure S2. Determination of the inhibitory effect of the thiosemicarbazide derivatives 1-10 in Topoisomerase IIα relaxation assay (compounds 11 and 12 were excluded from enzymatic tests because of their low solubility in the buffers used). Lane 1: scDNA; lane 2: scDNA + topoII; lane 3: scDNA + topoII + compound 1 (25µM); lane 4: scDNA + topoII + compound 1 (50µM); lane 5: scDNA + topoII + compound 1 (100µM); lane 6: scDNA + topoII + compound 2 (25µM); lane 7: scDNA + topoII + compound 2 (50µM); lane 8: scDNA + topoII + compound 2 (100µM); lane 9: scDNA + topoII + compound 3 (25µM); lane 10: scDNA + topoII + compound 3 (50µM); lane 11: scDNA + topoII + compound 3 (100µM); lane 12: scDNA + topoII + etoposide (50µM); lane 13: scDNA + topoII + etoposide (100µM); lane 14: scDNA + topoII + etoposide (150µM).

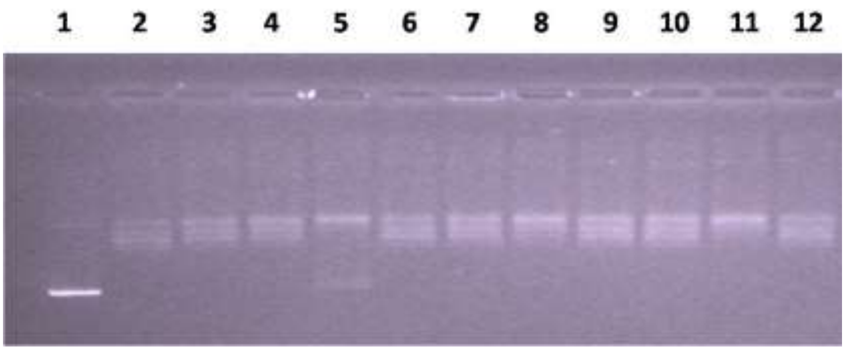

Figure S3. Lane 1: scDNA; lane 2: scDNA + topoII; lane 3: scDNA + topoII + etoposide (50µM); lane 4: scDNA + topoII + etoposide (75µM); lane 5: scDNA + topoII + etoposide (125µM); lane 6: scDNA + topoII + compound 4 (100µM); lane 7: scDNA + topoII + compound 5 (100µM); lane 8: scDNA + topoII + compound 6 (100µM); lane 9: scDNA + topoII + compound 7 (100µM); lane 10: scDNA + topoII + compound 8 (100µM); lane 11: scDNA + topoII + compound 9 (100µM); lane 12: scDNA + topoII + compound 10 (100µM).

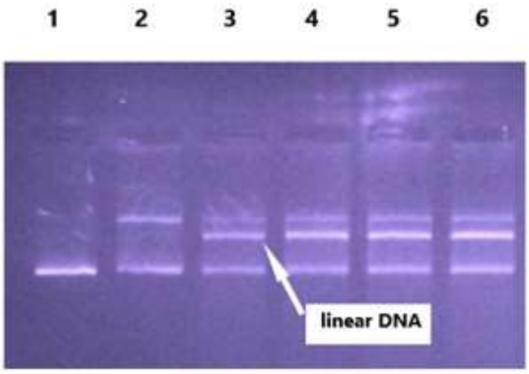

Figure S4. Stabilization of the „cleavable complex" by etoposide and compounds 1-3. Lane 1: scDNA; lane 2: scDNA + topoII; lane 3: scDNA + topoII + etoposide (50µM); lane 4: scDNA + topoII + compound 1 (50µM); lane 5: scDNA + topoII + compound 2 (50µM); lane 6: scDNA + topoII + compound 3 (50µM).
